# Large Area Deposition by Radio Frequency Sputtering of Gd_0.1_Ce_0.9_O_1.95_ Buffer Layers in Solid Oxide Fuel Cells: Structural, Morphological and Electrochemical Investigation

**DOI:** 10.3390/ma14195826

**Published:** 2021-10-05

**Authors:** Nunzia Coppola, Pierpaolo Polverino, Giovanni Carapella, Regina Ciancio, Piu Rajak, Montinaro Dario, Francesca Martinelli, Luigi Maritato, Cesare Pianese

**Affiliations:** 1 Dipartimento di Ingegneria Industriale DIIN, Università degli Studi and CNR SPIN, 84084 Fisciano, SA, Italy; lmaritato@unisa.it; 2 Dipartimento di Ingegneria Industriale DIIN, Università degli Studi di Salerno, 84084 Fisciano, SA, Italy; ppolverino@unisa.it (P.P.); pianese@unisa.it (C.P.); 3 Dipartimento di Fisica “E.R. Caianiello”, Università degli Studi di Salerno, 84084 Fisciano, SA, Italy; giocar@sa.infn.it; 4 Istituto Officina dei Materiali (IOM)-CNR, Laboratorio TASC, Area Science Park, S.S.14, Km 163.5, 34149 Trieste, Italy; ciancio@iom.cnr.it (R.C.); rajak@iom.cnr.it (P.R.); 5 Abdus Salam International Centre for Theoretical Physics, Via Beirut, 6, 34151 Trieste, Italy; 6 SOLIDpower S.p.A., 38017 Mezzolombardo, TN, Italy; dario.montinaro@solidpower.com (M.D.); francesca.martinelli@solidpower.com (F.M.)

**Keywords:** large area deposition, sputtering, sputtered buffer layer morphology, impedentiometric characterization

## Abstract

We investigate the influence of position, under large circular sputtering targets, on the final electrochemical performance of 35 mm diameter button solid oxide fuel cells with sputter-deposited Gadolinium doped Ceria barrier layers, positioned in order to almost cover the entirety of the area associated with a 120 × 80 mm^2^ industrial cell. We compare the results obtained via structural and morphological analysis to the Electrochemical Impedance Spectroscopy (EIS) measurements performed on the button cells, disentangling the role of different parameters. The Atomic Force Microscopy analysis makes it possible to observe a decrease in the roughness values from the peripheral to the central zones under the sputtering target, with peak-to-valley roughness values, respectively, decreasing from 380 nm to 300 nm, while Scanning Electron Microscopy and Energy Dispersive Spectroscopy show a dependence of the layer coverage from the position. The electrochemical performances of button cells with buffer layers of only 200 nm in thickness, and with negligible thickness gradients across them, show current density values of up to 478 mA/cm^2^ at 0.8 V and 650 °C, with an improvement of more than 67% with respect to button cells with standard (screen printed) buffer layers. These results point out the major influence exerted by parameters such as the thickness gradient and the coverage of the sputtered buffer layers in determining the final electrochemical performances.

## 1. Introduction

The lowering of the working temperature of Solid Oxide Fuel Cells (SOFC) down to the intermediate temperature (IT) range is, at present, crucial for the widespread use of this energy technology. The use of Doped Ceria (DC) barrier layers between the cathode and the electrolyte can improve the SOFC’ efficiency even if the working temperature lowers to the IT range (500 °C–700 °C) [1,2,3,4]. This temperature lowering is beneficial from the point of view of material durability and, consequently, in terms of the durability of the SOFC performance. Among all the different material synthesis techniques (e.g., screen printing, spin coating, etc.), Physical Vapour Deposition (PVD)-based techniques have recently attracted interest in relation to the production of DC barrier layers [5,6]. The use of a particular PVD technique, namely room temperature RF-Sputtering, followed by an optimized post-growth annealing, has been shown to ameliorate 35 mm-diameter SOFC button cells’ final performances when compared to cells with commercially screen-printed barrier layers [7,8]. Although other sputtering techniques [9,10,11] have been shown to produce DC buffer layers with good structural and electrical properties, the choice of room temperature RF sputtering is, in this case, justified by the very low thickness of the produced DC films and by the ease in scaling up sputtering processes without the need to heat large substrates during the deposition. Moreover, the fine tuning of the post annealing ramp’s temperature made it possible to control the final granularity barrier layer and, hence, the ratio between the number of grain boundaries versus the bulk part of the grain, yielding a further improvement in the electrochemical performance of the cells [8].

**Figure 1:**
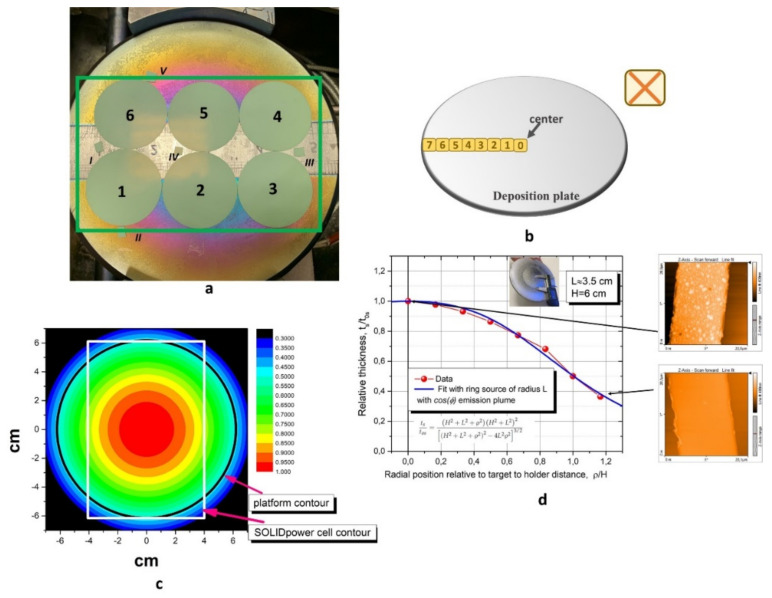
(**a**) View of the 35 mm in Ø half cells and control slivers with the nomenclature used in this paper superposed. (**b**) Schematic of the 8YSZ single crystals placed along the deposition plate’s radius; in the right corner, a schematic of the lithography cross geometry performed on each single crystal is displayed. (**c**) Contour plot of the radial distribution of thickness on the deposition plate obtained by performing AFM on the substrates after DC deposition. (**d**) Normalized thickness plotted as a function of the radial coordinate normalized to the target–substrate distance. In the inset, a target (here aluminium, previously mounted on same magnetron source as the actual DC target) showing an erosion profile that strongly peaked at the radius of a single ring source L = 3.5 cm; DC deposition.

The results in [7,8] opened the way for additional investigation of this technique in view of the possibility of scaling up to industrial production. Although, at present, the sputtering technique is largely used in many coating-related industrial processes, the area over which sputtered thin films display good homogeneity in terms of thickness and density is strongly dependent on the particular characteristics of the deposition system. In this work, we investigated, in detail, the influence that the specific sputtering procedure can have on the final performances of different zones of an industrial cell with dimensions 80 × 120 mm^2^. We adopted a simple approach in which the DC barrier layer was deposited on six half (anode plus electrolyte) button cells, which were distributed under the sputtering target in order to almost cover the entirety of the area associated with a standard industrial cell (see Figure 1a). The microstructure of the sputter-deposited DC layer was systematically studied by X-Ray diffraction (XRD) analysis, Atomic Force Microscopy (AFM), Scanning Electron Microscopy (SEM) and Energy Dispersive Spectroscopy (EDS). The comparison between the structural and morphological analysis and the Electrochemical Impedance Spectroscopy (EIS) measurement performed on the final cells with a screen-printed cathode made it possible to disentangle the role of different parameters, evidencing a major role played by the uniformity and coverage of DC thickness in determining the final cell performances.

## 2. Materials and Methods

The investigated samples were fabricated through the use of the magnetron Radio Frequency (RF)-Sputtering device (MRC RF-Sputtering, Electron Mec Company, Milan, Italy) based at the University of Salerno endowed with a 150 mm diameter target: Gd_0.1_Ce_0.9_O_1.95_; Testbourne 99.999% purity). The sputtering target dimension, the plasma active area, the target–substrate distance, the partial pressure of the operating gas (usually Ar) and the power applied to sustain the discharge are the parameters that typically influence the thickness and the density’s homogeneity during the sputtering process. In the case of the system used for this experiment, previous studies made it possible to select these parameter values in order to optimize the homogeneity and the density of the deposited DC layers on a 35 mm diameter circular SOFC [7,8]. The typical deposition process of the DC barrier layers used a 400 W power discharge in a 2.3 × $10^{-3}$ Torr Ar pressure. All the as-grown samples underwent an in air post-growth thermal treatment in a quartz tube furnace, using the temperature ramp reported in [8].

AFM analysis made use of a Nanite (Nanosurf, Liestal, Switzerland) equipped with a Tap190Al-G monolithic silicon Tip (Budget Sensors, Sofia, Bulgaria); measurements were performed in tapping mode with a 48 N/m constant force and 190 KHz resonance frequency. The AFM images had a 0.3 nm z resolution and a 1.5 nm xy resolution. Systematic XRD characterization was performed on as-grown and post-growth annealed sliver samples using a D2-Phaser Diffractometer (Bruker, Billerica, MA, USA). The diffractometer was endowed with a Cu-α radiation source characterized by a λ=1.541 Å wavelength; the angular resolution was set to 0.01 2θ degrees while the acquisition time was set to 0.4 s. All SEM images presented in the work were collected using a ZEISS SUPRA 40 high-resolution field emission gun (FEG) SEM (Zeiss, Oberkochen, Germany). The microscope was able to operate with a 4 pA–10 nA probe current and 0.1–30 kV electron high tension (EHT), with a nominal resolution of 1.3 nm at 15 kV. It was equipped with a Back-scattered Electron Detector (BSE), an Everhart–Thornley Secondary Electron (SE) Detector, and a High efficiency In-lens detector, the latter providing an increased signal-to-noise ratio in image acquisition. EDS measurements were performed using an Oxford Aztec energy dispersive X-ray spectroscopy (EDS) system annexed to the microscope consisting of an X-act 10 mm silicon drift detector (SDD) for compositional analysis. The performances of the cells with the sputtered DC layer were analysed by j-V and EIS measurements, using the test bench described in [7], after the fabrication of the complete 35 mm diameter fuel cells was achieved by screen-printing the cathodes [7]. The j-V and EIS measurements were carried out at three different cells’ working temperatures (650 °C, 700 °C and 750 °C) and, concerning EIS, two different voltages were explored (namely 800 and 900 mV). The gas feeding was provided with 16 Nl∙min^−1^∙cm^−2^ of hydrogen and 40 Nl∙min^−1^∙cm^−2^ of air. 

## 3. Results and Discussion

When depositing onto a large area, as in the case of standard industrial cells depositing on an area of 80 × 120 mm^2^, preliminary investigations about the thickness profile and uniformity over the whole surface interested in the process are required. We, therefore, initially placed eight 8YSZ single crystal substrates (SurfaceNet GmbH, Rheine, Germany), (111) oriented, 10 × 10 mm^2^ single-side polished) along the RF-Sputtering sample holder diameter, as shown schematically in Figure 1b, in order to investigate the radial distribution of the thickness. Through the use of photolithographic procedure, a suitable geometry, schematically sketched in the upper-right corner of Figure 1b, was obtained on each substrate, in order to allow direct measurement of the deposited thickness by means of AFM analysis.

The blue solid line, shown in Figure 1d, was the best fit of the data point obtained assuming that a relative radial distribution of thickness accounted for a single ring source of radius L with a standard $cos\left(\varphi \right)$ emission plume [12], which was in agreement with the formula reported in the figure (see Ref. [12]). Here, both the radial coordinate and the radius were normalized to the target to substrate distance H. The approximation of single radius was justified by the rather neat erosion profile of our magnetron, which peaked at approximately L = 3.5 cm, as can be appreciated in the inset of Figure 1d.

The observed 50% decrease in thickness going from the centre to the edge of the sample holder was expected in the case of a target–substrate distance of about 60 mm [12]. In Figure 1c, a contour colour plot of the measured thickness distribution is shown, along with the cell contour (white colour in the figure), evidencing the area covered by an 80 × 120 mm^2^ industrial cell. As it is clear from Figure 1c, the sputter deposition of a DC buffer layer on a cell implies a large DC thickness gradient along the cell, with thickness values on the peripheral zones halved with respect to those obtained in the central part. In view of possible industrial scaling up of the proposed DC deposition procedure, it is then crucial to understand how this thickness gradient and, more generally, the specific position under the sputtering target, can affect the overall final cell performances. We, therefore, performed a simulation experiment in which six 35 mm diameter anode-electrolyte half-cells were placed under the sputtering target, almost completely covering the area of an 80 × 120 mm^2^ cell (see Figure 1a). In Figure 1a, the roman numerals indicate the half-cell’s slivers placed alongside the button half-cells to make it possible to systematically perform morphological and structural analysis on indicative samples associated with different positions under the sputtering target.

The typical deposition time is 24 min for this procedure, according to previously estimated deposition rates [10], producing a final thickness of about 600 nm on the central zone under the sputtering target and about 300 nm of DC on the zones placed on the borders. 

A DC barrier layer was also deposited on a half button cell, labelled as cell 200, which was placed in the central zone under the sputtering target, in a different sputtering process in which the only changed parameter was the deposition time, which was lowered to eight minutes, in order to produce a 200 nm-thick DC layer. The post-growth annealing treatment was left unchanged.

### 3.1. Structural and Morphological Analysis

**Figure 2:**
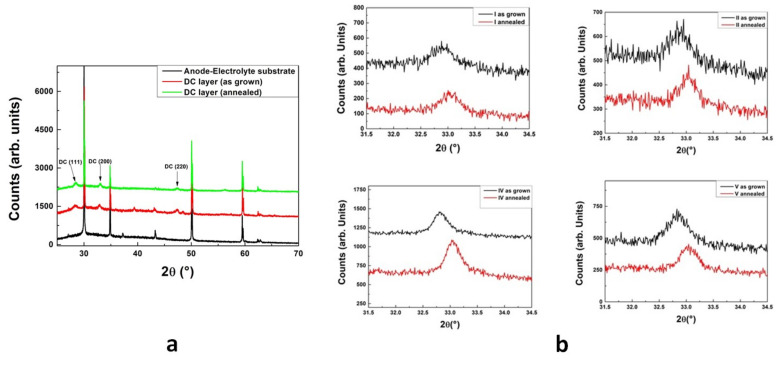
(**a**) XRD of the SOLIDpower anode-electrolyte substrate (black line) and of the deposited GDC layer, both as-grown (red line) and annealed (green line). Curves are vertically shifted for clarity. (**b**) (200) reflection XRD measurements for the slivers I, II, IV and V. For each of them, results regarding the as-grown sample (black line) and the annealed sample (red line) are displayed.

In Figure 2a, the XRD results obtained on sliver I, before the DC deposition (anode-electrolyte substrate), after the DC deposition (as-grown) and at the end of the annealing procedure (annealed), are shown. Intense peaks associated with the anode-electrolyte substrate were present in all the spectra. The peaks related to the desired DC phase (see Figure 2a) were already present in the as-grown samples and their intensity and angular position changed slightly during the annealing procedure.

In Figure 2b, the (200) reflections measured in four slivers (I, II, IV and V) are reported for both the as-grown and annealed samples, showing a slight shift toward larger angular positions. A similar shift was observed for all the DC reflections and, for all the as-grown samples, the value of the DC cubic lattice parameter, calculated from the angular position of the peaks, was found to be 5.44±0.01 Å, slightly larger than the expected bulk value [13], which was probably due to oxygen deficiency as a result of the room-temperature deposition process [14,15]. This oxygen deficiency was easily resolved by the post-growth annealing treatment, as displayed by the position shift of the peaks of the annealed samples in Figure 2b. For all the annealed slivers, the measured DC cubic lattice parameter value was 5.41±0.01 Å, which was in agreement with the bulk value [11]. The same results were obtained by XRD measurements performed on a sliver placed close to cell 200. From the XRD analysis, it is evident that the particular position of the cells under the sputtering target did not play a role in the obtainment of the desired stoichiometric phase for the DC buffer layers deposited on the top of the half button cells.

**Figure 3:**
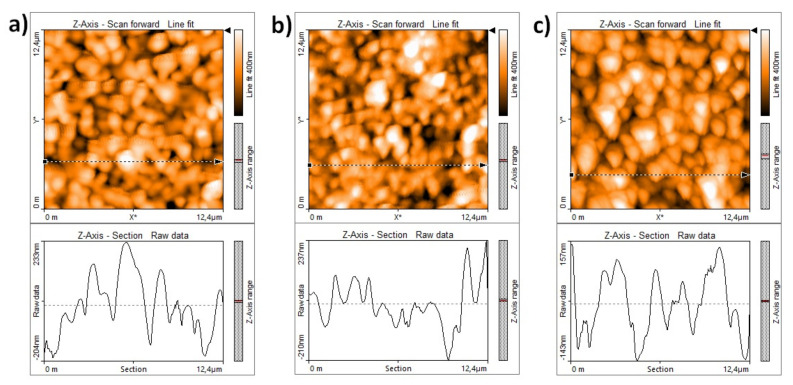
AFM images of (**a**) the anode-electrolyte substrate (R_z_ = 400 nm). (**b**) The as-grown cell 1 (R_z_ = 380 nm). (**c**) The as-grown cell 2 (R_z_ = 300 nm).

The surface morphology of the samples was investigated by means of AFM measurements. In Figure 3a, a typical AFM image of the surface of an anode-electrolyte half-button cell is shown with an estimated peak-to-valley roughness (R_z_) of around 400 nm and a root mean square average roughness (R_q_) of 100 nm. In Figure 3b,c, the AFM images taken on the surfaces of cell 1 and cell 2 after the sputter deposition of the DC layers are shown, respectively. As expected, the deposition of the DC buffer layer slightly decreased the surface roughness values with respect to those measured on the anode-electrolyte substrate. Moreover, we observed a decrease in the roughness values going from cell 1, placed far from the central zone, to cell 2, with a position closer to the centre of the sputtering target. In particular, cell 1 showed values of R_z_ and R_q_ of 380 nm and 80 nm, respectively, while for cell 2, the measured values were $R_{z}=300\;\mathrm{nm}\;\mathrm{and}\;\mathrm{Rq}=70\;\mathrm{nm}.$ The roughness values measured by AFM on the cells after the annealing procedure were the same as those observed in the case of the as-grown cells. AFM measurements performed on the as-grown cell 200 showed peak-to-valley and root mean square average roughness values that were somehow in between those of cell 1 and cell 2 ($R_{z}=340\;\mathrm{nm}\;\mathrm{and}\;\mathrm{Rq}=70\;\mathrm{nm},$ respectively. Generally, for sputtered thin films deposited on rough substrates, a surface roughness dependence upon the film thickness is expected, with decreasing roughness values for increasing thickness. We note that, in this case, the AFM analyses were performed on the 35 mm diameter cells. Due to the thickness profile shown in Figure 1c, a DC thickness gradient was present in all cells numbered from 1 to 6, but not in cell 200. All the AFM measurements were performed close to the centre of each cell, over an area where the DC thickness of cell 1 should be in the range 500–400 nm and that of cell 2 in the range of 600–500 nm. Therefore, by comparing the AFM results of cell 1 with those obtained in the case of cell 200, we deduced that, not only the total film thickness, but also its gradient across the cell and, more generally, the position of the cells under the sputtering target, influenced the morphology of the deposited DC layer, particularly when looking at the surface roughness of the sputtered films. From Figure 3b,c, it is also clear that there was an appreciable increase in the lateral (xy) dimensions of the grains going from cell 1 to cell 2, indicating a better tendency towards crystallization in the case of cell 2, which was probably due to its position being closer to the central region under the target where higher energy species were sputtered. Such a tendency was also clear in the AFM images in Figure 1d, relative to the DC layers deposited on YSZ single crystal substrates, and was confirmed by analysing the width of the DC XRD peaks of samples with similar thicknesses and different positions under the sputtering target.

To obtain complementary information on the composition and morphology of the sputter-deposited barrier layers, control silvers were inspected by Scanning Electron Microscopy (SEM) combined with Energy Dispersive Spectroscopy (EDS).

**Figure 4:**
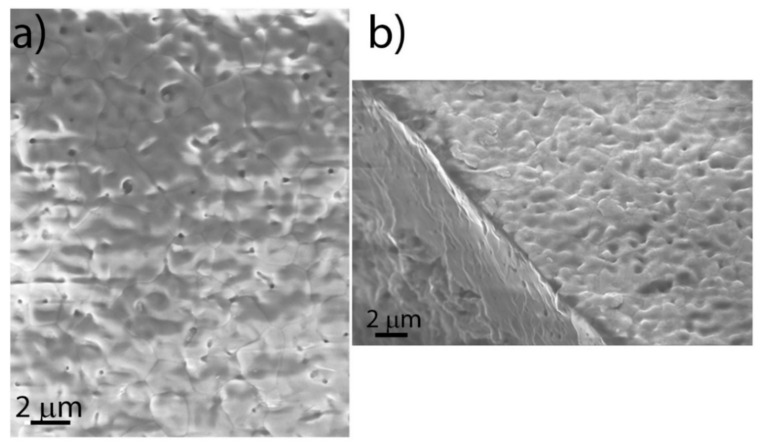
SEM images of the anode/electrolyte half-cell substrate provided by SOLIDpower acquired, before the deposition process, with the SE (**a**) in plan view and (**b**) in tilting condition.

**Figure 5:**
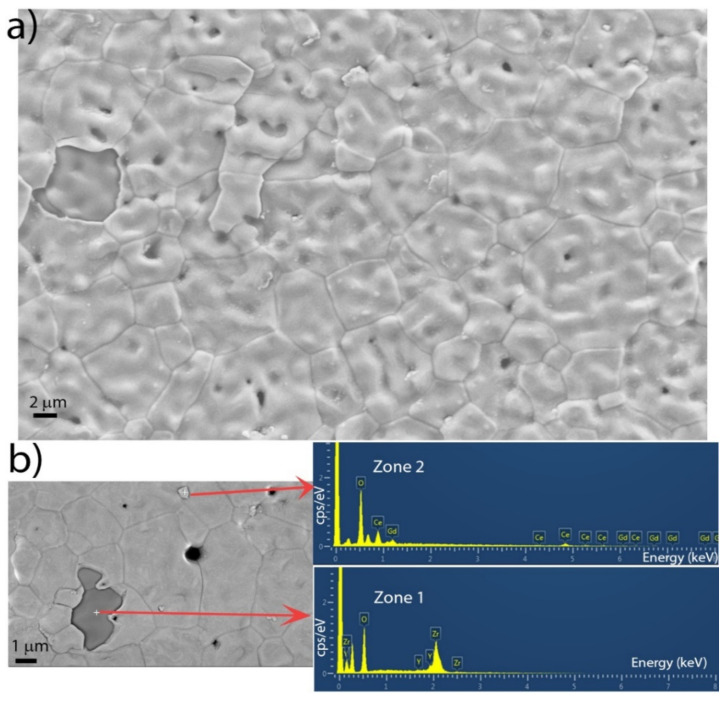
(**a**) SEM image performed on the sliver (sliver 200) placed alongside cell 200 with the SE detector. (**b**) EDS measurements on two different zones of sliver 200 (see text).

As a first step, we analysed the anode-electrolyte substrate. Representative SEM images taken with the SE detector in plan view and in tilting condition are shown in Figure 4a,b, respectively. The substrate had a surface morphology that was characterized by micrometre-sized plaquettes decorated with small holes. In Figure 5a, a SEM image taken on the sliver placed close to cell 200 (sliver 200 in the following) is displayed. The substrate appears to have been completely covered by the 200 nm DC layer, which reproduced the surface morphology of the buried substrate. To disentangle between the 200 nm DC layer and the substrate, we show, in Figure 5b, the EDS spectra taken across two different zones of the sample: one where a small piece of the DC layer had lifted off (zone 1) and another one that was entirely covered by the DC film (zone 2). The EDS spectrum of zone 1 only shows the characteristic peaks of the YSZ substrate, while in zone 2 the Gd and Ce peaks are also clearly visible. Through quantitative EDS, it was established that the Gd:Ce stoichiometric ratio was equal to 0.13:0.87, which was in good agreement, within the boundaries of experimental uncertainty, with our expectation.

**Figure 6:**
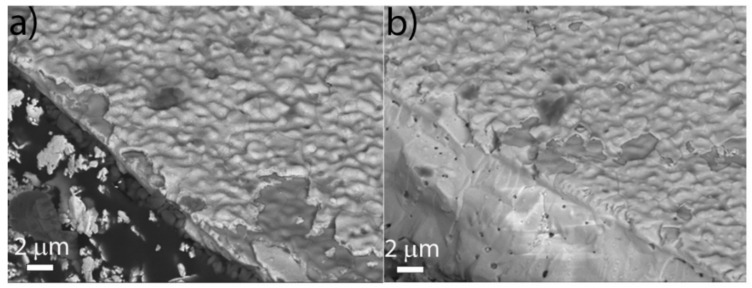
SEM images taken on (**a**) sliverIII and (**b**) sliver 200 by the use of the BSE detector.

In the Figure 6a,b, the SEM images taken on sliverIII and sliver 200, respectively, in zones close to the edges are shown. The images were taken with the BSE detector with tilting of the sample. In this condition, it was indeed easier to visualize the DC buffer layer and the bare substrate since they had slightly different contrasts due to their intrinsic chemical differences. Qualitatively, the observed surface roughness seemed to be in agreement with the AFM results, slightly increasing from sliverIII to sliver 200.

Sampling different zones of the investigated slivers using SEM, we observed a tendency to present larger areas without a good DC coverage when going from sliver 200 to sliverIV and sliverIII. The SEM analysis was performed on the samples a few months after the sputter deposition. The differences in the observed coverage, therefore, could have been related to the sample position under the sputtering target and, in particular, to the presence of different strains acting on the DC layers, which was likely due to differences in thickness and grain dimension. 

From all of the structural and morphological analyses performed on the cells and the slivers, we concluded that the desired DC phase was obtained in all of the samples independently of the specific position under the sputtering target. On the other hand, this position played a role in the observed surface roughness and buffer layer covering. 

### 3.2. Impedentiometric Measurements

**Figure 7:**
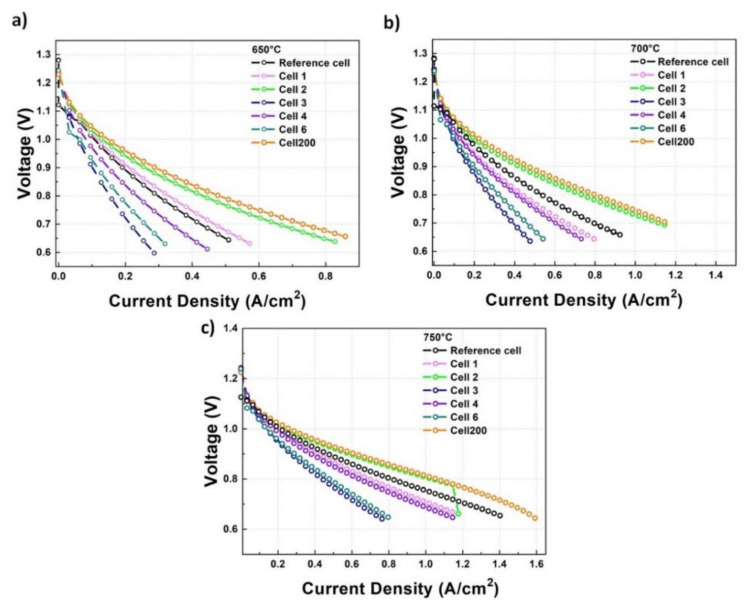
j-V curves at 650 °C (**a**), 700 °C (**b**) and 750 °C (**c**) for cell 1 (pink line), cell 2 (green line), cell 3 (blue line), cell 4 (violet line), cell 6 (dark cyan line) and cell 200 (orange line). The reference cell (black line) has a screen-printed barrier layer.

In Figure 7, the j-V results for cell 1–6, cell 200, and a reference cell with a screen-printed DC buffer layer, are shown at the three investigated temperatures.

As is clear from the figure, the relative performance of the cells differed considerably with current values taken at 0.8 and 0.9 V, varying by more than a factor of two. At all of the temperatures, among cells 1–6, cell 2 was the one with the best performances. At 750 °C, the j values of cells 3 and 6 were very close and were the lowest observed. By decreasing the working temperature, they continually showed the worst performances even though their respective j values were increasingly different, with cell 6 always performing better than cell 3. A similar behaviour was observed for cells 1 and 4, with j values of cell 1 always being higher than those obtained for cell 4. At all of the temperatures, the performances of cell 6 and cell 1 were better than those of cell 3 and cell 4, probably indicating a slight asymmetry, with respect to the centre, in the positioning of the cells and/or in the sputtered material flow. The performances of cell 4, cell 6 and cell 3 were always worse than those of the Reference cell. Cell 1 had j values that were slightly lower than those of the Reference cell at 700 °C and 750 °C, but showed higher values at 650 °C. Looking at the positions of the cells under the sputtering target, as shown in Figure 1a, we note that cells 3 and 6 and cells 1 and 4 were symmetrically located with respect to the centre of the sample holder. Their similar behaviours were, therefore, not surprising. Moreover, the spreading of the j-V curves for these cells with the lowering of the temperatures can be explained in terms of the role played by the YSZ electrolyte layer. In fact, regarding the final performances of the cells, the relative importance of the YSZ electrolyte with respect to the DC buffer layer was expected to increase with the temperature. At higher temperatures, therefore, the cells were expected to perform more similarly; the observed results were in agreement with this expectation. On the other hand, at lower temperatures, the role of the DC buffer layers starts to become more important and the observed differences in the performance of the cells should be related to different properties of the sputtered DC films. As outlined in the previous section, the surface roughness and coverage of the DC-sputtered layers depended upon the specific cell position under the sputtering target, while the same DC crystallographic phase was obtained in all the cells. The observed differences in the measured j-V curves of the analysed cells can, therefore, be tentatively traced back to differences in their structural and morphological properties. Due to their position, looking to the thickness contour in Figure 1c, the investigated cells were expected to have different DC layer thickness with different thickness gradients. In particular, cell 1,3, 4 and 6 were expected to have thicknesses in the range of 500–300 nm, while cell 2 was expected to present thickness values in the range of 600–500 nm. To investigate the influence of these parameters on the final performances of the cells, we also analysed the electrochemical properties of cell 200, which were expected to present a uniform thickness of 200 nm all over its surface. As is clear from the data in Figure 7a, the j-V curves measured for cell 200 always presented the best performances, with the j-V curves of cell 2 showing only slightly lower values than those of cell 200. This is a strong indication that, in achieving the best electrochemical performances, a major role is played by the thickness uniformity and coverage, and not by the surface roughness and the absolute thickness value. We reiterate that the surface roughness measured on cell 2, in the previous section, showed a value of 300 nm, which was lower than the one observed on cell 200 (340 nm), and that the thickness of cell 2 varied in the range of 600–500 nm. 

In Table 1, we report, in detail, the current density values measured at 800 and 900 mV on all of the samples at the three investigated temperatures. The %Δj values are defined as the ratio $\frac{j\left(\mathrm{cell}\;y\right)-j\left(\mathrm{Reference}\right)}{j\left(\mathrm{Reference}\right)}$.

We point out that, according to the data in Table 1, cell 2 and cell 200 had %Δj values of +56% and 67% respectively, at 800 mv and 650 °C.

The electrochemical characterization was completed by performing Electrochemical Impedance Spectroscopy (EIS) measurements at the same set of three different working temperatures, at 800 and 900 mV.

**Figure 8:**
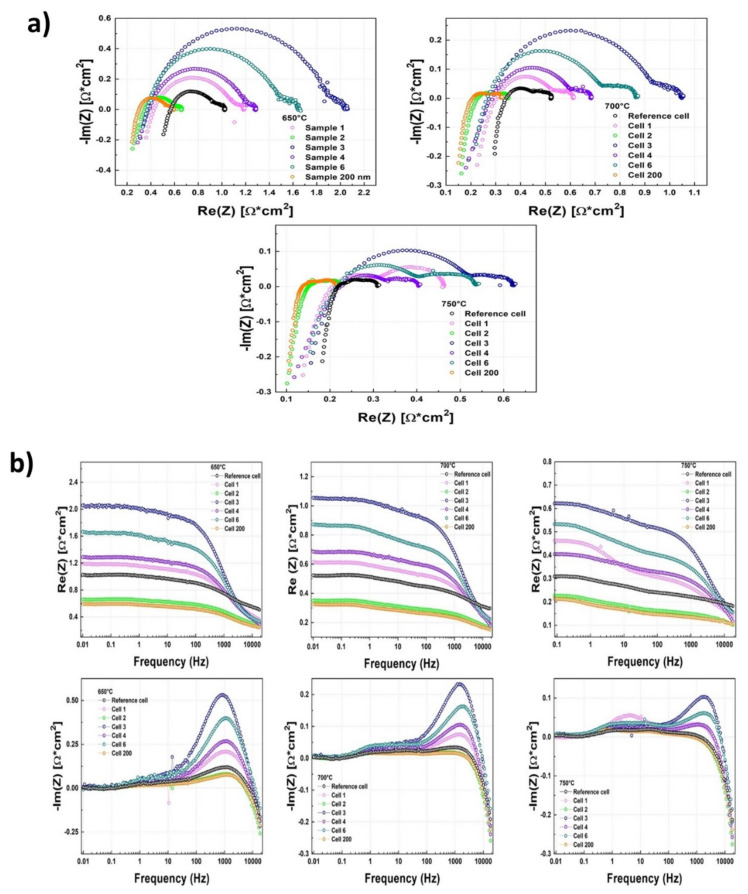
Nyquist (**a**) and Bode (**b**) plots at 650 °C, 700 °C and 750 °C for cell 1 (pink line), cell 2 (green line), cell 3 (blue line), cell 4 (violet line), cell 6 (dark cyan line) and cell 200 (orange line). The reference cell (black line) has a screen-printed barrier layer.

In the following, as an example, the Nyquist plots for each sample at 900 mV, as shown in Figure 8a, are displayed for different temperatures.

Using the data contained in the Nyquist plots, we calculated the high (R_HF_) and low frequency (R_LF_) resistances along with the polarization resistances (R_P_) (evaluated as the differences between the low and high frequency intercepts). The results are reported in Table 2, Table 3 and Table 4 in the case of the values obtained at 900 mV. 

As expected, the values of R_HF_ measured on the cells with DC-sputtered buffer layers were generally lower than those on the REF cells, due to the overall reduction in thickness. The values of R_LF_ and R_P_ instead followed the behaviour already observed in the j-V curves. Similar behaviours were also observed in the Nyquist plots at 800 mV. In Figure 8b, the Bode plots for all of the samples, at 900 mV, are shown at all of the investigated temperatures. 

Looking at the Bode plots of the impedance’s modulus, we obtained further confirmation of the behaviours already observed in the case of the j-V curves, with cell 2 always showing a lower value of the impedance modulus when compared with the reference sample, while the other cells were always characterized by higher values. On the other hand, for the impedance’s phase, we note the presence of a remarkable increase in the phase’s values at high frequencies, in the case of cell 1, 3, 4 and 6, while this was not observed in cell 2 and cell 200. This increase, more pronounced at lower temperatures, was generally associated with inductive effects, and again, its observed occurrence in cells with less uniform thickness and coverage could be traced back to more disordered paths followed by the charged species when passing through the cell, particularly in the DC buffer layer. Similar results were also observed in the Bode plots at 800 mV.

The analysis of the j-V and EIS measurements, when compared with the results of the structural and morphological characterization, reveal that the specific position under the sputtering target, in the large deposition area investigated, influenced the final performance of the cells. In particular, our experimental results indicate that a major role was played by the uniformity and coverage of the DC thickness, while the surface roughness and absolute thickness values of DC seemed to have minor importance in relation to the determination of the electrochemical cell properties. The observed results are of relevance in terms of the further industrial scaling up of this technique. In particular, the minor role played by the absolute thickness value opens up the possibility of important decreases in the deposition process time.

## 4. Conclusions

The room temperature RF-sputter deposition of the DC barrier layer in solid oxide fuel cells, over a large area, was investigated by positioning six half button cells under a 150 mm diameter sputtering target in order to almost cover the entirety of the area associated with a standard industrial cell. The cells with sputter-deposited DC layers were structurally and morphologically characterized using XRD, AFM, SEM and EDS measurements. The electrochemical properties of the cells were analysed using j-V and EIS measurements. From the structural and morphological analysis, we observed that, while the DC stoichiometric phase was equally present in all of the cells, their surface roughness, thickness and thickness gradient varied as a function of their position. The cross comparison of the structural and morphological investigation with the electrochemical results provided further evidence of the influence of the cells’ position under the sputtering target on their final performances. In particular, by also analysing the results obtained on a cell with a DC layer of only 200 nm thickness, we were able to disentangle the role played by the different parameters in the final performance of the cells. The gradient and coverage of the DC thickness seemed to be the major aspects that influenced the electrochemical properties, while the surface roughness and the thickness appeared to play only minor roles. These results represent important insights in view of the industrial scaling up of the investigated deposition technique and, in particular, for the design of a specific industrial sputtering system dedicated to the SOFC production.

## Figures and Tables

**Table 1:** Current density values j for each of the examined samples for each working temperature with a comparison with a reference sample (Reference).

Sample	T = 750 °C		T = 700 °C		T = 650 °C	
	V = 900 mV		V = 900 mV		V = 900 mV	
	j (mA/cm^2^)	%Δj (%)	j (mA/cm^2^)	%Δj (%)	j (mA/cm^2^)	%Δj (%)
Reference	478	0	319	0	191	0
Cell 1	414	−13.4	255	−20.1	223	+16.7
Cell 2	573	+19.9	414	+29.8	255	+33.5
Cell 3	255	−46.7	159	−50.2	104	−45.5
Cell 4	382	−20.1	255	−20.1	147	−23.0
Cell 6	287	−39.9	191	−40.1	114	−40.3
Cell 200	604	+26.4	478	+49.8	287	+50.3
**Sample**	**V = 800 mV**		**V = 800 mV**		**V = 800 mV**	
	**j (mA/cm^2^)**	**%Δj (%)**	**j (mA/cm^2^)**	**j (mA/cm^2^)**	**%Δj (%)**	**j (mA/cm^2^)**
Reference	828	0	541	0	286	0
Cell 1	700	−15.5	445	−17.7	318	+11.2
Cell 2	1018	+22.9	732	+35.3	445	+55.6
Cell 3	445	−46.3	286	−47.1	154	−46.1
Cell 4	636	−23.2	414	−23.5	236	−17.5
Cell 6	478	−42.3	319	−41.0	183	−36.0
Cell 200	--	--	794	+46.8	478	+67.1

**Table 2:** High frequency intercept values (R_HF_) evaluated for each examined sample at 900 mV and 750 °C, 700 °C and 650 °C, and corresponding %ΔR_HF_ values calculated in comparison with the reference cell.

Sample	T = 750 °C		T = 700 °C		T = 650 °C	
	V = 900 mV		V = 900 mV		V = 900 mV	
	R_HF_ (mΩ∙cm^2^)	%ΔR_HF_ (%)	R_HF_ (mΩ∙cm^2^)	%ΔR_HF_ (%)	R_HF_ (mΩ∙cm^2^)	%ΔR_HF_ (%)
Reference	219	0	343	0	568	0
Cell 1	204	−6.8	302	−11.9	430	−24.3
Cell 2	149	−31.9	225	−34.4	343	−39.6
Cell 3	226	+3.2	273	−20.4	374	−34.1
Cell 4	227	+3.7	286	−16.6	393	−30.8
Cell 6	215	−1.8	265	−22.7	366	−35.6
Cell 200	137	−37.44	207	−39.6	302	−46.8

**Table 3:** Low frequency intercept values (R_LF_) evaluated for each examined sample at 900 mV and 750 °C, 700 °C and 650 °C, and corresponding %ΔR_LF_ values calculated in comparison with the reference cell.

Sample	T = 750 °C		T = 700 °C		T = 650 °C	
	V = 900 mV		V = 900 mV		V = 900 mV	
	R_LF_ (mΩ∙cm^2^)	%ΔR_LF_ (%)	R_LF_ (mΩ∙cm^2^)	%ΔR_LF_ (%)	R_LF_ (mΩ∙cm^2^)	%ΔR_LF_ (%)
Reference	313	0	526	0	1029	0
Cell 1	463	+47.9	608	+15.6	1181	+12.9
Cell 2	229	−26.8	349	−36.2	656	−36.2
Cell 3	623	+99.0	1048	+99.2	2049	+99.1
Cell 4	403	+28.7	682	+29.7	1289	+25.3
Cell 6	537	+71.6	870	+65.4	1659	+61.2
Cell 200	214	−31.6	323	−38.6	594	−42.3

**Table 4:** Polarization resistance values (R_P_) evaluated for each examined sample at 900 mV and 750 °C, 700 °C and 650 °C, and corresponding %ΔR_P_ values calculated in comparison with the reference cell.

Sample	T = 750 °C		T = 700 °C		T = 650 °C	
	V = 900 mV		V = 900 mV		V = 900 mV	
	R_P_ (mΩ∙cm^2^)	%ΔR_P_ (%)	R_P_ (mΩ∙cm^2^)	%ΔR_P_ (%)	R_P_ (mΩ∙cm^2^)	%ΔR_P_ (%)
Reference	94	0	183	0	461	0
Cell 1	259	+175.5	306	+67.2	751	+66.9
Cell 2	80	−14.9	124	−32.2	313	−32.1
Cell 3	397	+322.3	775	+323.5	1675	+263.3
Cell 4	176	+87.2	396	+116.4	896	+94.3
Cell 6	250	+165.9	679	+271.0	1545	+235.1
Cell 200	77	−18.1	116	−36.6	292	−36.7
